# A Rare Case of Heterozygous Gain of Function Thyrotropin Receptor Mutation Associated with Development of Thyroid Follicular Carcinoma

**DOI:** 10.1155/2018/1381730

**Published:** 2018-10-17

**Authors:** James Blackburn, Dinesh Giri, Barbara Ciolka, Nicole Gossan, Mohammad Didi, George Kokai, Alison Waghorn, Matthew Jones, Senthil Senniappan

**Affiliations:** ^1^Department of Paediatric Endocrinology, Alder Hey Children's NHS Foundation Trust, Liverpool, UK; ^2^Department of Histopathology, Alder Hey Children's NHS Foundation Trust, Liverpool, UK; ^3^Merseyside and Cheshire Regional Genetics Laboratories, Liverpool Women's Hospital, Liverpool, UK; ^4^Department of Endocrine Surgery, Royal Liverpool Hospital, Liverpool, UK; ^5^Department of Paediatric Surgery, Alder Hey Children's NHS Foundation Trust, Liverpool, UK; ^6^Institute of Child Health, University of Liverpool, Liverpool, UK

## Abstract

Activating mutations in thyrotropin receptor (*TSHR*) have been previously described in the context of nonautoimmune hyperthyroidism and thyroid adenomas. We describe, for the first time, a mutation in* TSHR *contributing to follicular thyroid carcinoma (FTC) in an adolescent. A 12-year-old girl presented with a right-sided neck swelling, increasing in size over the previous four weeks. Clinical examination revealed a firm, nontender thyroid nodule. Ultrasound scan of the thyroid showed a heterogeneous highly vascular mass. Thyroid function tests showed suppressed TSH [<0.03mU/L], normal FT4 [10.1pmol/L, 9-19], and raised FT3 [9.1pmol/L, 3.6-6.4]. Thyroid [TPO and TRAB] antibodies were negative. A right hemithyroidectomy was performed and the histology of the sample revealed follicular carcinoma with mild to moderate nuclear pleomorphism and evidence of capsular and vascular invasion (pT1b). Sanger sequencing of DNA extracted from the tumour tissue revealed a missense somatic mutation (c.1703T>C, p.Ile568Thr) in* TSHR*. Papillary thyroid carcinomas constitute the most common thyroid malignancy in childhood, while FTC is rare. FTC due to* TSHR *mutation suggests an underlying, yet to be explored, molecular pathway leading to the development of malignancy. The case is also unique in that the clinical presentation of FTC as a toxic thyroid nodule has not been previously reported in children.

## 1. Introduction

Thyroid carcinoma is a rare diagnosis in paediatric population. The most common form of thyroid carcinoma in children and adolescents is papillary carcinoma; the incidence of follicular carcinoma in children is less than 10% of all thyroid cancers [1]. The clinical features of follicular carcinoma are highly variable but typically include expanding neck mass, compression of local structures, and subtle features such as behavioural changes. Clinical diagnosis of FTC can be challenging, as there remains no single method for differentiating follicular adenoma and follicular carcinoma. The use of fine needle aspiration of a thyroid mass can be considered; however presence of cytology of indeterminate significance including follicular proliferation and atypia of undetermined significance in between 5 and 20% of cases limits the use of this method [2]. In such patients, firm diagnosis is often made by histological analysis of frozen sections from intraoperative thyroid masses. Treatment involves removal of the primary lesion and to ensure complete eradication, radioactive iodine therapy would be often required.

Given the relatively low prevalence of follicular carcinoma, evidence in terms of treatment and outcomes is limited. The role of genetics in the development of this cancer is also poorly understood. We report, for the first time, a follicular carcinoma in a 12-year-old girl due to gain of function mutations in* TSHR.*

## 2. Case Report

**Figure 1:**
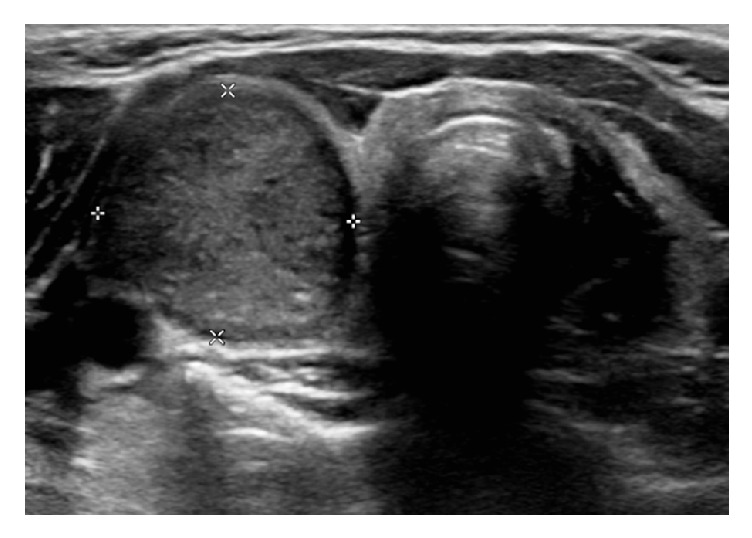
Thyroid ultrasound scan shows a round well circumscribed heterogeneous, highly vascular mass arising from the right lobe of the thyroid, measuring 21 x 17 x 17 mm.

The patient is a 12-year-old Caucasian girl referred urgently to the endocrinology clinic with an expanding right neck mass. The mass had first been noted four weeks prior to their appointment and was felt to have increased in size during this time. Examination revealed a well-grown prepubertal girl with no clinical features suggestive of hyper- or hypothyroidism. On examination of the neck, a firm right sided neck mass was noted. This measured 2 cm x 1.5cm and was not tethered to any local structures. An urgent thyroid ultrasound scan revealed a round well circumscribed heterogeneous, highly vascular mass arising from the right lobe of the thyroid, measuring 21 × 17 x 17 mm (Figure 1). No lymphadenopathy was noted. Chest X-ray was normal with no evidence of mediastinal lesion or lung mass. The thyroid function test showed raised FT3 (9.1pmol/L [normal range 3.6-6.4]) and normal FT4 (free T4 10.1pmol/L [normal range 9-19]), with suppressed TSH (<0.03mU/L [normal range 0.3-3.8]). After a detailed discussion with the family, hemithyroidectomy was undertaken for removal of the lesion.

**Figure 2:**
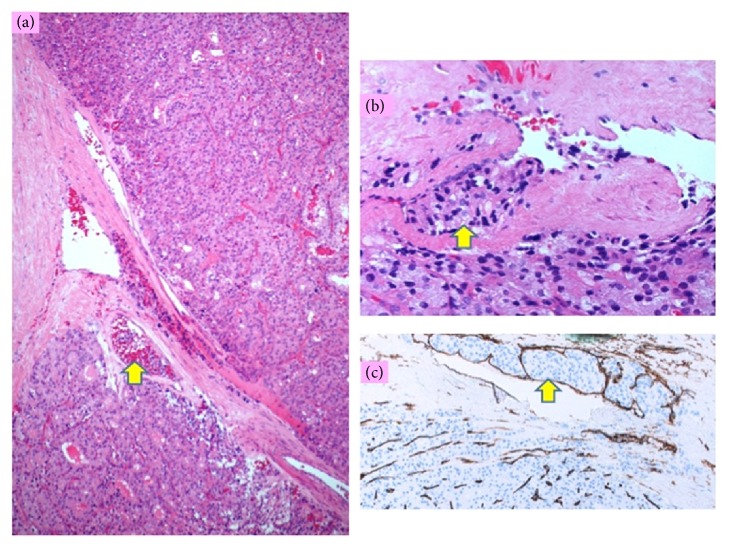
(a) Tumour and a capsule with a vessel containing tumour cells, arrow (H&E, x80); (b) tumour cells aggregate within the vessel lumen of the capsule (HE, x360); (c) intravascular tumour cell aggregate covered by endothelial cells (CD34, x100).

Macroscopic examination of the surgical specimen showed a well circumscribed 20 mm mass. Microscopic examination of the specimen showed a predominately insular and follicular growth pattern. There were no features of papillary nuclear changes or anaplastic component. Mild to moderate nuclear pleomorphism with some mitotic features were noted (Figure 2). A diagnosis of follicular thyroid carcinoma (pT1b) was made and the patient underwent completion thyroidectomy. Histological examination of the extracted left thyroid gland showed benign thyroid tissue with no evidence of residual carcinoma.

The majority of the tumour showed follicular and compact growth pattern with only few areas of more lobular appearance, although the typical insular growth was not present. The vascular invasion was limited to only four small caliber vessels (veins) within the capsule (two illustrated on the submitted images) and the capsular invasion affected 3/4 of its thickness without actually penetrating it. The insular thyroid carcinoma is rare (from 0.3 to 6.7% of all thyroid cancers) and mainly affects adults >45 years of age, although there are isolated case reports in young children [3]. Given the tumour cells were predominantly well differentiated, follicular carcinoma was confirmed as the diagnosis. This has been confirmed by expert review at the time of the hemithyroidectomy.

Levothyroxine was commenced postoperatively with normalisation of the thyroid function [TSH 1.7, FT4 11.9pmol/L]. Corrected calcium [2.38 mmol/L, normal range 2.15-2.74 mmol/L] and PTH [5.9pmol/L, normal range 1.1-6.9pmol/l] were stable during the postoperative period.

Three months after thyroidectomy, the patient received a course of radioactive iodine. Whole body scan showed no evidence of distant metastases. The patient is currently on thyroxine 125 micrograms once daily and the thyroid function is normal.

The Sanger sequencing of the DNA extracted from the tumour tissue revealed a missense* TSHR *mutation (c.1703T>C, p.Ile568Thr). The mutation was present with a frequency of 25% within the sample, representing a somatic gain of function mutation.

## 3. Discussion

The diagnosis of thyroid carcinoma is relatively rare in paediatric population. Recent research suggests the differentiation of paediatric thyroid tumours which were classified as papillary (60%), follicular variant of papillary (23%), follicular (10%), and medullary (5%) [1]. The most common presentation is with a gradually expanding neck mass. Patients younger than 15 years old at diagnosis are more likely to have more extensive tumour at diagnosis than patients who were 15 years and above [4]. Conventional FTCs virtually never involve regional lymph nodes and have distant metastases, most commonly to the lungs and bones (10–20% of cases) [5].

Our patient was unique in that the presentation of a FTC is rarely associated with a toxic thyroid nodule. Paediatric patients are occasionally found to have an autonomously functioning nodule (toxic adenoma) diagnosed by a suppressed TSH and increased nodule-specific uptake on nuclear medicine radioisotope scan (^99m^Tc pertechnetate or iodine-123 [^123^I]). These lesions could be associated with somatic activating mutations within the genes encoding the TSH receptor or the G_s_-alpha subunit. On examination, children either are euthyroid or may have mild signs or symptoms of hyperthyroidism [6]. The current recommendation for treatment of autonomous nodules is surgical resection, due to the increased risk of malignancy, particularly in paediatric population [7]. Survival rates are 98% for minimally invasive FTC and 80% for invasive follicular carcinoma [8].

A hyper functioning thyroid nodule associated with papillary carcinoma has previously been described [9]. The patient had clinical features of hyperthyroidism, which was confirmed by biochemical and functional imaging. Analysis of the excised tissue showed evidence of papillary carcinoma. Molecular studies showed evidence of met453thr mutation in* TSHR*.

The development of FTC from a toxic nodule has previously been described in adults [10], where somatic mutations of* TSHR* were reported; however to our knowledge this is the first described presentation of FTC from a toxic thyroid nodule in a paediatric patient.

Thyrotropin receptor [TSHR] is a member of the seven transmembrane-spanning receptors, one of the subfamilies of glycoprotein hormone receptors (GPHR) [11]. Somatic mutations in both G proteins and G-protein coupled receptors have recently been implicated in up to 20% of all human cancers [12].* TSHR* mutations are associated with multiple cancers including thyroid, lung, and ovarian although the role of TSHRs in these cancers remains unclear [13]. Mutations in RAS and the PAX8/PPAR*γ* rearrangement have been implicated in adult FTC; however the somatic genetic events that contribute to the pathogenesis of paediatric FTC remain largely unstudied [14, 15]. Adult studies have suggested that the 5th–6th transmembrane domains and the 3rd extracellular loop regions of the* TSHR* have the highest frequency of activating mutations [16].

**Figure 3:**
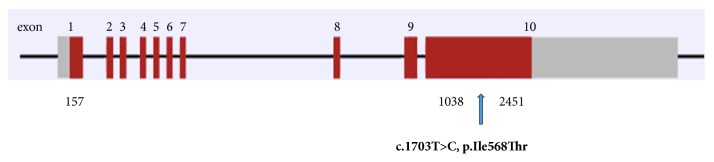
Schematic representation of* TSHR* containing 10 exons (numbered 1-10 in red) with the introns represented by the horizontal line joining the exons. The grey region represents the untranslated region (UTR). The position of the variant at the position 1703 is shown in exon 10.

The genetic abnormality described in our patient (c.I703T>C) was a heterozygous gain of function thyrotropin receptor mutation. A substitution of cytosine to thymine was detected, changing isoleucine 568 into a threonine (I568T) (Figure 3). This was a somatic mutation given the frequency of mutation in the sample tissue.

A somatic mutation of* TSHR* gene (c.I703T>C) has been previously described in a patient who presented with a toxic thyroid adenoma and was demonstrated to have potent activation of the adenylate cyclase pathway [16]. The mutation has also been shown to be significantly activating in vitro with elevated levels of cyclic adenosine monophosphate. The increased cellular signalling is independent of TSH stimulation [17]. Increased levels of inositol phosphate have also been shown to be present in vitro in cells which carry the ile568thr mutation [18]. The subsequent cellular pathways that have been associated with development of FTC in this patient remain as yet unclear but clearly represent an opportunity for further research.

Although mutation has been described previously in association with toxic thyroid adenomas, its association with development of FTC has not been previously described. Review of* TSHR* mutation database did not reveal a previously described association with the development of FTC [19].

A similar mutation has been described previously by Tonacchera et al. [20]; however this was a germline mutation with a heterozygous* TSHR* detected by PCR gene amplification. The patient described in the case developed an acute neonatal hyperthyroidism, with associated goitre, on day fifteen of life. The patient was treated on day forty-four with propylthiouracil and propranolol. Following establishment of euthyroidism, dose tapering led to a recurrence of a clinical hyperthyroidism [20]. No behavioural, neurological, or motor defects were noted in long-term follow-up. Similar cases have also been reported in association with nonautoimmune neonatal hyperthyroidism [21], with thyroid tissue masses reported after therapeutic thyroidectomy [22].

The genetic basis of follicular carcinoma in paediatric patients is still to be well understood. The genetic basis of thyroid cancers has largely been focused upon the genetic basis of papillary thyroid carcinoma [23]. Recent studies, which have sought to further characterise genetic mutations of FTC, have used whole exome sequencing of tumour samples to identify associated genetic mutations. In addition to* TSHR *mutations, commonly associated mutations include* NRAS, HRAS, BRAF,* and* EIF1AX *[24]. The collective mutation rates between follicular adenoma and carcinoma are similar to most gene mutations noted in both cell types.* TSHR *mutations were also reported in both cell populations. The generalisation of these results to paediatric patients is limited as the sequencing was only performed on adult type tissue samples.

## 4. Conclusion

We describe, for the first time, FTC due to* TSHR *mutation suggesting an underlying, yet to be explored, molecular pathway leading to the development of malignancy. The case also highlights the unusual presentation of FTC, in this case arising from a toxic thyroid nodule. Similar cases have been described in adults; however to our knowledge this is the first such presentation in a paediatric patient.
